# Clinical Characteristics of Persistent Hypophosphatasemia Uncovered in Adult Patients: A Retrospective Study at a Japanese Tertiary Hospital

**DOI:** 10.3390/jcm13237078

**Published:** 2024-11-23

**Authors:** Shintaro Fujiwara, Yuki Otsuka, Masanori Furukawa, Akihito Higashikage, Fumio Otsuka

**Affiliations:** 1 Department of General Medicine, Okayama University Graduate School of Medicine, Dentistry and Pharmaceutical Sciences, Okayama 700-8558, Japan; fujiwarashintaro1@gmail.com (S.F.); otsuka@s.okayama-u.ac.jp (Y.O.); 2 Department of Pediatrics, NHO Okayama Medical Center, Okayama 701-1192, Japan; 3 Department of Laboratory Medicine, Okayama University Hospital, Okayama 700-8558, Japan

**Keywords:** chronic fatigue syndrome, chronic pain, hypophosphatasemia, alkaline phosphatase, hypophosphatasia

## Abstract

**Background:** Hypophosphatasemia is often overlooked despite its potential to indicate underlying pathologies. The aim of this study was to determine the prevalence of persistent hypophosphatasemia in a large, urban, multi-specialty hospital population and characterize the clinical and laboratory findings in adult patients with this condition. **Methods:** In this retrospective observational study, the results of 424,434 alkaline phosphatase (ALP) tests in 50,136 patients aged ≥18 years that were performed at Okayama University Hospital between July 2020 and October 2023 were analyzed. Persistent hypophosphatasemia was defined as consistently low ALP levels (≤40 IU/L) for 28 days with a minimum recorded level of ≤35 IU/L. **Results:** Persistent hypophosphatasemia was detected in 273 patients (0.54% of the tested patients), and the patients with persistent hypophosphatasemia included a higher proportion of females (72.5% vs. 52.9% in the people without persistent hypophosphatasemia; chi-squared test, *p* < 0.01) and had a younger median age (51 years vs. 63 years; Mann–Whitney U test, *p* < 0.01) than those in the overall tested population. The common causes of persistent hypophosphatasemia were cancer (30%), glucocorticoid use (21%), and immunosuppressants (16%). Notably, 38 patients (14%) had no apparent cause for low ALP values. These patients were categorized on the basis of their clinical characteristics, with some patients presenting symptoms potentially related to adult hypophosphatasia. **Conclusions:** This study provides prevalence and insights into the causes and characteristics of persistent hypophosphatasemia in a Japanese tertiary care setting. While most cases were associated with known causes, patients with unexplained hypophosphatasemia and symptoms such as chronic pain, muscle weakness, and general fatigue could have adult hypophosphatasia. In such cases, comprehensive evaluation and further investigation for hypophosphatasia should be considered. Persistent hypophosphatasemia of undetermined etiology could be a crucial initial step in diagnostic algorithms for this condition.

## 1. Introduction

Alkaline phosphatase (ALP) is a membrane-bound enzyme that is responsible for releasing phosphate groups in the human body [1]. It has four major isozymes, each coded by distinct genes [2]. Among them, the *ALPL* gene encodes tissue nonspecific alkaline phosphatase (TNSALP), which constitutes the largest portion of total serum ALP. TNSALP is primarily expressed in the liver, bones, and kidneys, though it is also present in smaller amounts in other tissues [2].

In healthy adults, serum ALP predominantly consists of bone and liver isoforms of TNSALP [3]. An elevated serum ALP level is a well-established biochemical marker for diagnosing various liver and bone disorders. However, conditions associated with hypophosphatasemia are less common and often unrecognized, as no diagnostic algorithm exists, although the conditions may also indicate important underlying pathologies [4,5].

Various diseases and treatments can result in hypophosphatasemia, and hypophosphatasia (HPP), a genetic metabolic bone disorder, is one of the most well-known causes. HPP is caused by the loss of function mutations in the *ALPL* gene that encodes TNSALP [6]. TNSALP plays a crucial role in skeletal and dental mineralization. A principal function of TNSALP is the catalysis of inorganic pyrophosphate hydrolysis to inorganic phosphate. In patients with HPP, TNSALP deficiency results in elevated inorganic pyrophosphate concentrations, leading to compromised mineralization of osseous and dental tissues [6]. HPP ranges in severity and symptoms, with six major categories classified by the age of onset and clinical manifestations: perinatally severe, perinatally benign, infantile, childhood, adult, and the dental-specific type as odontohypophosphatasia.

In adults, HPP may either represent late-onset manifestations of cases diagnosed in childhood or mild cases with less severity that passed unrecognized [7]. Adult HPP often presents less dramatically, making its prevalence uncertain. Nevertheless, the quality of life in adult patients with HPP is significantly reduced due to chronic bone pain, muscle pain, joint pain, and gait disturbance [8]. Misdiagnosis of HPP as osteoporosis is not uncommon, and treatment regimens for osteoporosis, such as bisphosphonates, can exacerbate symptoms of HPP [9,10]. Although HPP is rare, its correct diagnosis is critical. Diagnosing HPP can be challenging due to its nonspecific symptoms [8]. Consequently, several studies propose genetic analysis for patients with hypophosphatasemia, regardless of symptom presentation, as a potential means to aid in diagnosing adult HPP [11,12,13,14]. However, conducting such testing for all patients with hypophosphatasemia in daily practice is not feasible. In these previous investigations, genetic analyses were conducted on patients with hypophosphatasemia, defined by each study protocol. Although several studies attempted to exclude cases of hypophosphatasemia with identifiable etiologies, the exclusion criteria seemed to be insufficient. Moreover, detailed clinical manifestations in patients with unknown causes of hypophosphatasemia have not been adequately characterized.

Persistent hypophosphatasemia is a useful screening tool for HPP, and identifying the clinical characteristics and underlying causes in patients with hypophosphatasemia can help to determine if genetic testing is warranted. Despite the importance of this approach, studies focusing on clinical profiles of patients with hypophosphatasemia are scarce, and, to our knowledge, no report on adults has been published in Japan. Since adult HPP presents with nonspecific symptoms as described above, it is crucial to remain vigilant for low ALP levels in routine practice and to recognize the characteristics of these patients to avoid missing a potential diagnosis.

The aim of this retrospective study was to determine the prevalence of persistent hypophosphatasemia in a large, urban, multi-specialty hospital population and to characterize the clinical and laboratory findings in adult patients with persistent hypophosphatasemia

## 2. Materials and Methods

### 2.1. Study Design and Patients

This retrospective observational study included patients aged ≥18 years who underwent ALP testing at Okayama University Hospital, a large, tertiary care, multi-specialty hospital in Okayama Prefecture, Japan. All ALP measurements from patients aged ≥18 years were included, encompassing both outpatient and inpatient setting across all of the hospital departments. This hospital serves as a referral center for complex medical cases from across the region and beyond. The study period extended from July 2020 to October 2023. A total of 424,434 ALP tests were performed on 50,136 patients in the study period.

### 2.2. Method for Measuring Serum ALP Levels

Over the four-year period from 2020 to 2023, ALP tests were performed using the JEOL Bio Majesty™ JCA-BM8040 analyzer equipment and a commercially available assay kit (Pure-Auto S ALP-IFCC, Sekisui Medical Co., Tokyo, Japan). The enzymatic method used to assess the activity is the colorimetric method recommended by the International Federation for Clinical Chemistry (IFCC). The reference normal range of ALP levels in Japanese adults is 38–113 IU/L by the IFCC method. The lower limit of the range varies slightly from 35 to 40 IU/L, depending on sex and age.

### 2.3. Definition of Persistent Hypophosphatasemia

**Figure 1:**
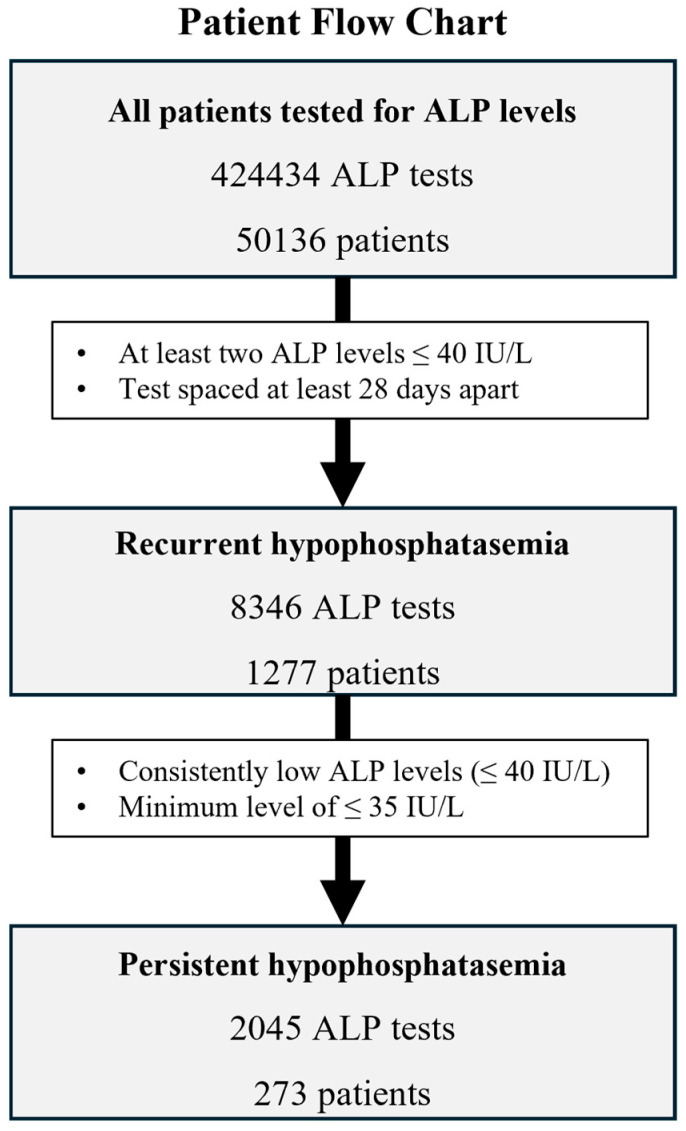
Flow chart of selection of patients. Of 424,434 patients being tested for serum ALP, 1277 were identified with recurrent hypophosphatasemia, and 273 of those patients were identified as having persistent hypophosphatasemia based on consistently low ALP levels (≤40 IU/L) and a minimum recorded level of ≤35 IU/L.

Figure 1 shows the patient flow chart. Among the 50,136 patients, 1277 patients were found to have recurrent hypophosphatasemia, defined as having ALP levels ≤40 IU/L in at least two tests with an interval of at least 28 days. Of this group, 273 patients were identified as having persistent hypophosphatasemia, defined by consistently low ALP levels (≤40 IU/L) and a minimum recorded level of ≤35 IU/L. The criterion of persistent hypophosphatasemia in this study was defined based on previous reports to investigate hypophosphatasemia [5,7,11,14,15]. Previous studies predominantly adopted ALP levels of ≤40 IU/L to define hypophosphatasemia, with one study employing a cutoff of ≥45 IU/L for exclusion. In this study, we established a similar threshold of ≤40 IU/L used in previous reports but implemented more stringent criteria to enhance the accuracy of identifying persistent hypophosphatasemia. These criteria stipulated that patients must (1) have never exhibited ALP levels >40 IU/L and demonstrate persistently low ALP levels (≤40 IU/L), and (2) record at least one measurement ≤35 IU/L. The minimum ALP level of ≤35 IU/L was set to exclude patients with ALP values within the normal range, as the lower limit of the normal range for Japanese adults varies from 35 to 40 IU/L by age and sex.

### 2.4. Data Collection

For each patient with persistent hypophosphatasemia, demographic, clinical, and laboratory data (white blood cells, hemoglobin, platelets, albumin, aspartate aminotransferase, alanine aminotransferase, γ-glutamyl transferase, blood urea nitrogen, creatinine, estimated glomerular filtration rate, calcium, phosphorus, magnesium, zinc, C-reactive protein (CRP), thyroid-stimulating hormone, and free thyroxine) were extracted from electronic medical records. This study exclusively utilized electronic medical records to ensure standardized data collection, accurate temporal tracking of ALP measurements, and comprehensive access to clinical information, including medical histories, medications, and concurrent diagnoses. The underlying causes of hypophosphatasemia were also thoroughly evaluated, and the researcher collected narrative data to identify potential causes of low ALP levels and information on medical history and clinical symptoms. To minimize the risk of oversight, when multiple factors were present in a single patient, all relevant contributors were considered. The causes of hypophosphatasemia, as defined by previous reports [5,7,11,14,15], are shown in Supplementary Table S1. Cancer-related hypophosphatasemia was defined as low ALP occurring during cancer treatment (chemotherapy, immunotherapy, surgery, or palliative care) or in patients with diagnosed but untreated cancer. Hypophosphatasemia was not considered cancer-related in patients with a history of cancer who had completed treatment, were disease-free during the study period, and were in good general health.

### 2.5. Statistical Analysis

Categorical data are presented as numbers and frequencies. Continuous variables are presented as medians and interquartile ranges (IQRs). We hypothesized that patients with persistent hypophosphatasemia might be younger and exhibit minimal sex differences. Age and sex distribution of patients with persistent hypophosphatasemia were compared to those without it. Since the age data were not normally distributed, the nonparametric Wilcoxon rank-sum test was employed for comparison. Statistical significance was defined as a two-sided *p*-value < 0.05.

In addition, post-hoc power analysis was performed. According to the results of the chi-square test, the power of the study was found to be 0.99, with an effect size of 0.3 and an alpha error probability of 0.05. The sample size of the persistent hypophosphatasemia group (n = 273) was sufficient according to these values. All statistical analyses except for power analysis were conducted using R (version 4.4.1 (15 June 2024 ucrt); Copyright © 2024; The R Foundation for Statistical Computing, Platform: ×86_64-w64-mingw32/×64). Post-hoc power analysis was performed using G Power software (version 3.1.9.7) for Windows (University of Düsseldorf) [16].

### 2.6. Ethics Approval

This study was approved by the Research Ethics Review Committee of our institution (registration number 2401-014). The requirement for informed consent from the patients’ guardians was replaced with an opt-out process due to the retrospective nature of this study. All procedures were performed in accordance with the Declaration of Helsinki and its amendments.

## 3. Results

### 3.1. Summary of All ALP Measurements

**Figure 2:**
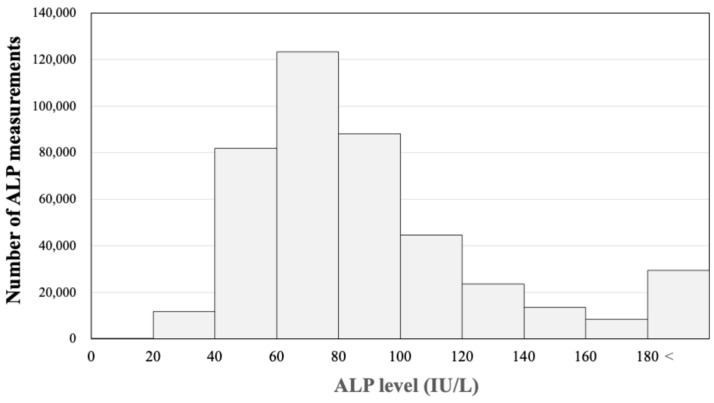
Histogram of all measured ALP levels in this study. The highest frequency of ALP values was in the range of 60 to 80 IU/L, with fewer tests as values deviated from this range. Abnormally high ALP levels (greater than 180 IU/L) accounted for 56,381 (13.3%) of the measurements. Additionally, 11,791 tests (2.77%) showed ALP levels of ≤40 IU/L and 8575 tests (2.02%) showed ALP levels of ≤38 IU/L.

A total of 424,434 serum ALP measurements were included in this study. Figure 2 shows the histogram of all measured ALP levels in this study. The highest frequency of ALP values was in the range of 60 to 80 IU/L, with fewer tests as values deviated from this range. Abnormally high ALP levels (greater than 180 IU/L) accounted for 56,381 (13.3%) of the measurements. Additionally, 11,791 tests (2.77%) showed ALP levels of ≤40 IU/L and 8575 tests (2.02%) showed ALP levels of ≤38 IU/L. In Japan, the normal range, encompassing 95% of healthy Japanese adults, is defined as 38–117 IU/L based on the Japanese Committee for Clinical Laboratory Standards. This suggests that the percentage of tests showing low ALP levels in the current study is slightly lower than what is typically observed in healthy Japanese adults.

### 3.2. Characteristics of Patients with Persistent Hypophosphatasemia

**Figure 3:**
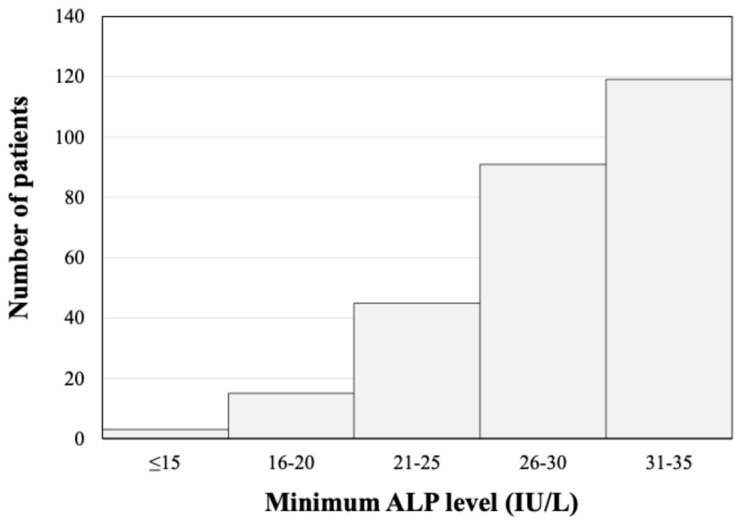
Histogram of the number of patients with persistent hypophosphatasemia. Lower minimum ALP levels were associated with fewer patients.

In the present study, 273 patients with a total of 2045 ALP measurements were identified as having persistent hypophosphatasemia. The minimum ALP values for the patients are shown as a histogram in Figure 3, which indicates that lower ALP levels were associated with fewer patients. Table 1 summarizes the sex and age distributions of patients with persistent hypophosphatasemia compared to all of the patients who had ALP levels measured in this study. The data indicated a higher proportion of females among those with persistent hypophosphatasemia than that in all patients (72.5% vs. 53.1%) and a younger median age of patients with persistent hypophosphatasemia (51 years, IQR 39–69) than that in all patients (62 years, IQR 44–74). Table 2 shows the laboratory results for patients with persistent hypophosphatasemia. All of the values were within the normal ranges except for C-reactive protein levels.

### 3.3. Causes of Persistent Hypophosphatasemia

Table 3 shows the causes of persistent hypophosphatasemia in the patients. The most common cause was cancer (n = 83, 30%), followed by the use of glucocorticoids (n = 56, 21%), immunosuppressants (n = 43, 16%), antiresorptive drugs (n = 39, 14%), hormone therapy (n = 36, 13%), and surgery (n = 35, 13%). Regarding cancer type, breast cancer was the most common type of cancer (n = 28, 34%), followed by gastrointestinal cancer (n = 17, 20%), urological cancer (n = 16, 19%), and gynecological cancer (n = 9, 11%). Most of the glucocorticoids and immunosuppressants were used for the treatment of autoimmune disorders, as shown in Supplementary Tables S2 and S3 (glucocorticoids: n = 42, 75%, immunosuppressants: n = 40, 93%). Apart from cancer, most of the apparent causes were treatment-related. Despite a detailed review of the patients’ medical records, 38 patients (14%) had no clear causes of persistent hypophosphatasemia.

### 3.4. Categorization of Persistent and Uncertain Hypophosphatasemia

Table 4 summarizes the age, sex, minimum ALP values, diagnosis, history, and clinical characteristics of the 38 patients without apparent causes of persistent hypophosphatasemia. In these patients, there were more females than males (31 females and 7 males). Based on their clinical characteristics, the patients were divided into six categories. Category 1 included nine patients with a history of cardiovascular disease: two patients had undergone transcatheter closure for an atrial septal defect, two patients had ablation therapy for paroxysmal tachycardia, and four patients had surgery for congenital heart disease in early childhood, and one patient had untreated paroxysmal ventricular tachycardia since childhood. None of those patients required treatment or exercise limitation and were only followed up as outpatients. Category 2 included six young female patients undergoing orthognathic surgery for malocclusion. Those patients had persistently low ALP levels before and long after the procedure, with no association with a temporary decrease related to surgery. Category 3 included 12 patients with chronic fatigue syndrome or muscle, bone, and joint pain in the extremities. All of those patients underwent a screening blood test for thyroid and adrenal endocrine functions, as well as collagen disease, resulting in no diagnosis. Category 4 included one patient who underwent endoscopic pituitary surgery and three patients who had thyroid glands. None of those patients exhibited endocrinological abnormalities, and they did not require replacement therapy. Categories 5 and 6 included a few patients with urological diseases who had normal renal function and those with obesity or hypertension. Three patients could not be categorized: one patient had a history of surgery for lung cancer, another patient had a history of stomach cancer treated with endoscopic submucosal dissection, and the third patient had a splenic cyst.

## 4. Discussion

### 4.1. Prevalence of Persistent Hypophosphatasemia

In this retrospective observational study, which included 424,434 ALP measurements from 50,136 patients, we examined the characteristics of patients with persistent hypophosphatasemia who presented with various symptoms. To ensure accuracy, we applied a strict definition of persistent hypophosphatasemia, excluding patients with transient hypophosphatasemia. Consequently, a total of 273 patients (0.54% of all tested patients) with 2045 ALP test results were identified, accounting for 0.48% of all test results. This suggests that approximately 0.5% of the general population tested for ALP at our hospital have persistent hypophosphatasemia, whether recognized or not. Additionally, 38 patients, accounting for 0.076% of all tested patients, had no apparent cause for low ALP values in our cohort. This prevalence is consistent with recent previous investigations, which reported rates of unexplained persistent hypophosphatasemia in the adult population of 0.06% [5], 0.10% [11], and 0.05% [12] in their respective study population.

### 4.2. Age, Sex, and Laboratory Characteriscs of Patients with Persistent Hypophosphatasemia

Notably, a significant sex difference was observed among patients with persistent hypophosphatasemia, even though no such difference was seen in the overall group of patients tested for ALP levels. There are two main reasons for this discrepancy. First, women are more likely to have underlying diseases that cause persistent hypophosphatasemia and often require treatment that results in a decrease in ALP level. Autoimmune disorders, which are more common in females after middle age, frequently necessitate long-term corticosteroid therapy and immunosuppressive therapy, both of which can lead to persistent hypophosphatasemia.

Glucocorticoids exert potent anti-inflammatory effects, suppressing the production of cytokines involved in inflammatory responses and inhibiting ALP production in osteoblasts and the liver [17]. Additionally, glucocorticoids are believed to impair osteoblast differentiation and promote apoptosis by directly affecting bone metabolism pathways, which further reduces ALP activity [18,19]. Similarly, immunosuppressive drugs, particularly calcineurin inhibitors such as cyclosporine and tacrolimus, impair osteoblast differentiation and inhibit bone formation, contributing to decreased ALP activity. For instance, cyclosporine A reduces the expression of osteoprotegerin in osteoblasts and bone marrow-derived stromal cells, consequently diminishing bone formation and lowering ALP levels [20,21].

Hypothyroidism, including Hashimoto’s thyroiditis, is another recognized cause of persistent hypophosphatasemia and is more common in females. Furthermore, breast cancer, predominantly affecting females, was the most common cancer associated with persistent hypophosphatasemia in this study, and its specific contributions will be discussed below. In fact, when comparing the proportions of patients with persistent hypophosphatasemia across all departments, the highest ratio was observed in the Department of Rheumatology, followed by Breast Oncological Surgery.

Secondly, the lower normal range of ALP levels varies slightly by sex and age, with females generally having slightly lower levels than males. According to the Japanese Society of Clinical Chemistry (reference ranges for major clinical laboratory tests in Japan by the Japanese Committee for Clinical Laboratory Standards [22]), the lower normal limit for all Japanese adults is 38 IU/L. However, when stratified by sex and age, the lower normal limits are 35 IU/L for premenopausal females, 38 IU/L for postmenopausal females, and 40 IU/L for males. This age-related difference in ALP levels may explain why younger female patients more frequently had low ALP values. To account for this, we defined persistent hypophosphatasemia in this study as ALP levels below 35 IU/L to exclude premenopausal females with normal ALP values. The laboratory results for patients with persistent hypophosphatasemia showed that, aside from CRP, all parameters were within normal ranges. This suggests that the study population was generally unbiased in terms of laboratory findings, including serum calcium and inorganic phosphorus, except for serum ALP and CRP levels. The elevated CRP levels observed in a small number of patients are likely attributable to inflammatory reactions, as this population consisted of individuals receiving hospital care.

### 4.3. Characteristics of Underlying Conditions of Persistent Hypophosphatasemia in Clinical Practice

Cancer was the leading cause of persistent hypophosphatasemia in the patients, with breast cancer being the predominant type, followed by digestive, urological, and gynecological cancers. Although breast cancer is not the most prevalent cancer at our hospital, it appeared most frequently in this study. We believe that there are two reasons for this. First, while cancer generally affects overall health and nutrition, it is possible that breast cancer itself may specifically contribute to a reduction in ALP levels [23,24]. Second, treatment-related factors, such as the use of selective estrogen receptor modulators (SERMs) and chemotherapy, are known to lower ALP levels. SERMs are often used for a long period to prevent recurrence after breast cancer surgery. Tamoxifen, in particular, a commonly used SERM, has been reported to inhibit bone turnover, resulting in reduced ALP levels in breast cancer patients. Similar effects have also been observed with other drugs [25,26,27,28]. In our cohort, 24 of the 28 patients with breast cancer were undergoing hormone therapy with SERMs. The high incidence of hypophosphatasemia in patients with breast cancer likely stems from a combination of disease characteristics and treatment effects.

Most non-cancer causes of persistent hypophosphatasemia were treatment-related, including the use of corticosteroids and immunosuppressants for autoimmune disorders. Notably, the use of glucocorticoids and immunosuppressants was for the treatment of autoimmune disorders in about 80% of the cases. Drugs for osteoporosis treatment, such as antiresorptive drugs, were frequently co-administered with corticosteroids, further contributing to ALP reduction. Several factors often overlapped in patients, particularly in those with autoimmune disorders, for whom steroids, immunosuppressants, and osteoporosis medications were used concurrently. Similarly, cancer patients, especially those with breast cancer, often had overlapping treatments, including surgery, chemotherapy, and SERMs. Independent causes, such as zinc deficiency and hypothyroidism, accounted for a fraction of the cases. In summary, cancer and autoimmune disorders were the most common underlying disorders, with treatment-related factors playing a significant role in reducing ALP levels. Despite extensive investigation, the cause of hypophosphatasemia remained unidentified in 38 patients. However, those patients were grouped into several categories based on their medical history.

### 4.4. Insights into Characteristics of Persistent Hypohphosphatasemia by Categorization

Category 1: Patients with a history of cardiovascular disease. While these individuals had normal cardiac function for years and did not require ongoing treatment, a potential link between heart disease and reduced ALP has been suggested in previous reports. Temporary declines in ALP levels have been observed in post-cardiac surgery, with greater reductions correlating to poorer outcomes [29]. Reduced cardiac output has also been linked to lower ALP levels in congenital heart diseases, such as Fontan circulation, though no such cases were present in this cohort [30]. However, no patients in Category 1 had Fontan circulation, and, to our knowledge, no association between decreased ALP levels and catheter closure for atrial septal defects or previous ablation, as seen in our study, has been reported. Future research may reveal associations as more cases are accumulated.

Category 2: Six patients who underwent orthognathic surgery. These patients had persistent hypophosphatasemia before and after surgery without a temporary decline due to the procedure. They had maxillary and mandibular malocclusion, but neither condition has been directly linked to HPP in previous reports. However, early loss of deciduous teeth, a possible symptom of HPP, can contribute to malocclusion [31,32]. Due to the retrospective nature of this study, information on detailed histories of premature tooth loss and fractures was not obtained for the patients. Approximately 400 variants of the ALPL gene have been identified [15], with the clinical significance of many still being unclear, and the symptom spectrum of adult HPP is also broad and not fully understood. Given these factors, although these patients may not exhibit typical symptoms of adult HPP, a detailed inquiry into other possible symptoms should be performed. If necessary, additional tests, such as pyridoxal phosphate (PLP) measurements and gene analysis, may provide new insights into the symptoms of adult HPP.

Category 3: Twelve patients in this category experienced muscle, bone, and joint pain in the extremities or back, as well as muscle weakness. Over half of these patients were diagnosed with myalgic encephalomyelitis/chronic fatigue syndrome (ME/CFS), a condition characterized by persistent extreme tiredness. Although the etiology of ME/CFS remains elusive, it can affect multiple body systems. Patients with CFS or those complaining of chronic pain in this category were prescribed symptomatic herbal and other medications. Interestingly, since ME/CFS is partly associated with elevated ALP levels [33], the presence of persistent hypophosphatasemia along with chronic pain and muscle weakness in these patients suggests adult HPP as a potential diagnosis.

Adult HPP is thought to be a relatively mild form of the condition and is usually diagnosed after middle age, manifesting through symptoms such as chronic pain, gait disturbances, stress fractures, and osteomalacia [34]. While the exact prevalence of adult HPP is uncertain, it has been estimated to range from 1 in 500 to 1 in 6000 individuals [35]. Additionally, a study in which whole-genome sequencing in 1322 healthy Japanese adults was analyzed identified the c.529 G > A variant, a possible pathogenic variant of adult HPP, with an allele frequency of 0.0107, associated with significantly lower serum ALP levels [36]. It should be noted that the presence of a genetic variant does not necessarily indicate the need for treatment, as many individuals with *ALPL* heterozygous variants and low ALP levels may remain asymptomatic. However, in cases such as in the present study in which low ALP levels persist and the etiology of chronic limb pain or general fatigue is unclear, HPP should be considered. In such cases, we would suggest that comprehensive clinical evaluation and further investigation, including genetic analysis, should be warranted. This diagnostic approach may facilitate the identification of previously undiagnosed adult HPP cases and prevent potential misdiagnoses such as osteoporosis. Previous genetic analyses of adults with persistently low ALP levels demonstrated *ALPL* variants in approximately 80% of cases, emphasizing the importance of careful diagnostic consideration before establishing a diagnosis of osteoporosis [13].

Recently, the international working group on HPP has developed diagnostic criteria for adult HPP. The guideline recommends patients with a persistently low ALP without any other identifiable cause. Certain characteristics, including the presence of chronic musculoskeletal pain, are suggested to be used as major and minor criteria for early diagnosis [37].

Category 4: One patient with a postoperative pituitary adenoma and three patients with nonfunctioning thyroid nodules or cysts. While thyroid hormones influence bone metabolism, ALP is typically elevated in hyperthyroidism and reduced in hypothyroidism. In these cases, thyroid hormone levels remained stable, and no clear association with ALP levels was found. Some patients with urological disease and some patients with metabolic disease were assigned to Categories 5 and 6, respectively. Due to the small number of patients in each group, with only two patients per group, it is difficult to determine a direct causal relationship between the disease and ALP reduction. For the three patients who did not align with any defined category, no notable similarities were identified among them.

In summary, patients in Category 3, especially those with chronic fatigue and pain, may harbor *ALPL* variants, raising the possibility of undiagnosed HPP. Further genetic testing and history reviews, including assessments for fractures or early tooth loss, are warranted to explore this connection.

### 4.5. Limitations and Strengths of This Study

This retrospective study has both strengths and limitations. This study represents the largest study in Japan on the characteristics of patients with persistent hypophosphatasemia, including a wide range of patients across hospital units and departments. A key strength of this study is its strict definition of persistent hypophosphatasemia, particularly for patients in whom ALP values never exceeded 40 IU/L, effectively excluding transient ALP reductions. However, several limitations exist. First, being a single-institution study introduces regional bias, although as a university hospital, it receives referrals from the community. As a tertiary care center, the patient population was relatively specialized, with a higher proportion of cancer patients and individuals with rare diseases. Also, as a referral medical institution, our center tends to treat patients with more severe or complex conditions compared to primary care facilities, where patients typically present with milder symptoms. This referral pattern may introduce potential selection bias in our study population. While this may limit generalizability, it also served as a strength, as it included a larger number of patients with complex complaints, particularly those in Category 3. Second, as a retrospective study, the presence of selection bias cannot be ruled out, potentially influencing the generalizability of the findings. In addition, not all potential causes of low ALP were comprehensively evaluated. Notably, serum zinc levels were only measured in a small subset of patients. Third, further study is needed to determine whether the current definition of persistent hypophosphatasemia is appropriate. Lastly, there was a lack of recognition of persistent hypophosphatasemia and awareness of HPP among both clinicians and patients. Therefore, even if symptoms or a medical history suggestive of HPP exist—such as a history of frequent bone fractures or early primary tooth loss—both the doctor and patient may overlook them. In view of this, we intend to analyze patients who fall into Category 3 by taking an individual medical history and measuring PLP levels. To address these limitations, future prospective studies should systematically evaluate patients with persistent hypophosphatasemia using interventional protocols to thoroughly investigate the factors contributing to decreased ALP levels, as well as symptoms or a medical history suggestive of HPP. Additionally, incorporating demographic and laboratory data from a control population would allow for statistical comparisons, helping to delineate the distinctive characteristics of persistent hypophosphatasemia. These analyses would enhance our understanding of the clinical and biochemical profile of this condition.

### 4.6. Conclusions

This study revealed the characteristics of patients with persistent hypophosphatasemia in a university hospital setting. Approximately 0.5% of the patients were affected, with a higher proportion of women and younger individuals compared to the general population. The primary causes of low ALP were treatment-related, including cancer therapies, steroid use, and the use of immunosuppressants. Notably, 0.076% of the patients had no known cause for their hypophosphatasemia, with some reporting chronic limb and bone pain. For these patients, the possibility of adult HPP was considered. Implementation of comprehensive clinical evaluation protocols in this subset of patients may facilitate early identification of adult HPP. The recognition of persistent hypophosphatasemia of undetermined etiology in daily clinical practice is very important because it serves as a crucial initial step in diagnostic algorithms for this condition.

## Figures and Tables

**Table 1:** Sex and age distributions of patients with and those without persistent hypophosphatasemia.

	All Patients	Persistent Hypophosphatasemia	Without Persistent Hypophosphatasemia	*p*-Value
	n = 50,136	n = 273	n = 49,899	
Age (years) ^a^	62 (44–74)	51 (39–69) ^b^	63 (53–72) ^b^	<0.01
Number and proportion (%) of the patients				
Female	26,670 (53.1%)	198 (72.5%) ^c^	25,833 (52.9%) ^c^	<0.01
Male	23,466 (46.9%)	75 (27.5%)	24,066 (47.1%)	

^a^ Data are expressed as medians and interquartile ranges (IQR); ^b^ Mann–Whitney U test; ^c^ chi-squared test.

**Table 2:** Laboratory data for patients with persistent hypophosphatasemia.

Result	Persistent Hypophosphatasemia ^a^	Reference Ranges ^b^
White blood cells ×10^9^ (L)	5.35 (4.53–6.82; n = 270)	3.3–8.6
Hemoglobin (g/L)	127 (117–134; n = 270)	
Platelets ×10^9^ (L)	215 (183–250; n = 270)	158–348
Alkaline phosphatase (IU/L)	30 (26–33; n = 273)	38–113
Albumin (g/L)	41 (38–43; n = 241)	41–51
Aspartate aminotransferase (IU/L)	18 (15–22; n = 272)	13–30
Alanine aminotransferase (IU/L)	13 (10–17: n = 273)	
γ-Glutamyl transferase (IU/L)	17 (13–26; n = 260)	
Blood urea nitrogen (mmol/L)	3.7 (4.8–6.1; n = 264)	2.9–7.1
Creatinine (µmol/L)	59 (54–60; n = 268)	
Estimated glomerular filtration rate (mL/min/1.73 m^2^)	74.6 (60.1–87.0; n = 268)	
Calcium (mmol/L) ^c^	2.3 (2.2–2.3; n = 202)	2.2–2.5
Phosphorus (mmol/L)	1.2 (1.0–1.3; n = 69)	0.9–1.5
Magnesium (mmol/L)	0.8 (0.8–0.9; n = 58)	0.7–1.0
C-reactive protein (µg/L)	0.06 (0.03–0.22; n = 260)	<0.03
Thyroid-stimulating hormone (mIU/L)	1.82 (1.13–3.30; n = 55)	0.35–4.94
Free thyroxine (pmol/L)	17 (14–18; n = 55)	9–19

^a^ Data are expressed as medians, interquartile ranges (IQRs), and numbers; ^b^ Reference range is defined to include the median 95% of healthy Japanese adults. (Collaborative derivation of reference intervals for major clinical laboratory tests in Japan by Japanese Committee for Clinical Laboratory Standards); and ^c^ For patients with albumin levels less than 4.0 g/L, the corrected calcium value calculated by the following formula was used.

**Table 3:** Underlying causes of persistent hypophosphatasemia.

Causes of Persistent Hypophosphatasemia	Number of Patients (Total: n = 273)	Percentage
Cancer	83	30%
Glucocorticoids	56	21%
Immunosuppressants	43	16%
Bone absorptive (bisphosphonates, denosumab)	39	14%
Selective estrogen receptor modulators	36	13%
Surgery	35	13%
Abnormal renal or liver function	29	10.60%
Clofibrates	26	9.50%
Hypothyroidism	24	9.20%
Chemotherapy	22	8.10%
Severe condition	16	6.20%
Gastrointestinal disease	6	2.20%
Malnutrition	1	0.36%
Vit.B12 deficiency	1	0.36%
Zinc deficiency	1	0.36%
Not related cause	38	14%

**Table 4:** Characteristics of patients without apparent causes of persistent hypophosphatasemia.

No.	Age	Sex	Lowest ALP	Diagnosis, History, and Clinical Characteristics of the Patients
Category 1: Patients with a history of cardiovascular disease				
1	76	male	21	a history of ablation therapy for paroxysmal tachycardia
2	74	male	31	a history of transcatheter closure for atrial septal defect
3	58	female	31	a history of ablation therapy for paroxysmal tachycardia
4	27	male	27	a history of transcatheter closure for atrial septal defect
5	23	female	33	a history of surgery for atrioventricular septal defect in early childhood
6	22	female	28	a history of surgery for pulmonary atresia with intact ventricular septum in early childhood
7	40	female	34	untreated paroxysmal ventricular tachycardia since childhood
8	24	female	29	a history of surgery for Tetralogy of Fallot in early childhood
9	23	female	30	a history of surgery for Tetralogy of Fallot in early childhood
Category 2: Patients who underwent orthognathic surgery				
10	31	female	26	a history of orthognathic surgery for malocclusion
11	23	female	27	a history of orthognathic surgery for malocclusion
12	47	female	29	a history of orthognathic surgery for malocclusion
13	42	female	30	a history of orthognathic surgery for malocclusion
14	32	female	30	a history of orthognathic surgery for malocclusion
15	19	female	35	a history of orthognathic surgery for malocclusion
Category 3: Patients with muscle, bone, and joint pain in the extremities or back, as well as muscle weakness				
16	39	female	29	chronic fatigue syndrome
17	35	female	35	chronic fatigue syndrome
18	28	female	34	chronic fatigue syndrome, muscle weakness in extremities
19	30	female	31	chronic fatigue syndrome, muscle weakness in extremities
20	30	female	34	chronic fatigue syndrome, muscle weakness in extremities
21	35	female	34	chronic fatigue syndrome, muscle weakness in extremities
22	88	female	31	joint pain in extremities, back pain
23	47	female	34	joint, muscle, and bone pain in extremities
24	40	female	33	general fatigue, muscle weakness in extremities
25	22	female	35	joint, muscle, bone pain, and muscle weakness in extremities
26	78	female	32	chronic fatigue syndrome, muscle pain in extremities
27	28	female	34	chronic fatigue syndrome, lack of focus
Category 4: Patients with asymptomatic endocrine disease				
28	47	female	32	a history of surgery for pituitary adenoma
29	33	female	28	nonfunctioning thyroid nodule
30	42	female	29	nonfunctioning thyroid nodule
31	61	female	32	nonfunctioning thyroid cyst
Category 5: Patients with urological disease				
32	44	female	24	chronic interstitial cystitis
33	33	male	24	ureteropelvic junction obstruction
Category 6: Patients with metabolic disease				
34	40	female	31	obesity, hypertension
35	34	male	17	obesity, hypertension, diabetes mellitus
Category 7: Others				
36	64	male	33	a history of surgery for lung cancer
37	67	male	34	a history of endoscopic submucosal dissection for stomach cancer
38	46	female	28	a splenic cyst with normal liver function

## Data Availability

The data that support the findings of this study are available on request from the corresponding author, F.O. The data are not publicly available; they contain information that could compromise the privacy of research participants.

## Supplementary Materials

The following supporting information can be downloaded at https://www.mdpi.com/article/10.3390/jcm13237078/s1; Table S1: Possible causes of known hypophosphatasemia; Table S2: Diseases and number of cases that required corticosteroid use; Table S3: Diseases and number of cases that required immunosuppressant use.

## Abbreviations

Alkaline phosphatase (ALP); hypophosphatasia (HPP); International Federation for Clinical Chemistry (IFCC); selective estrogen receptor modulators (SERMs); myalgic encephalomyelitis/chronic fatigue syndrome (ME/CFS); and tissue-nonspecific alkaline phosphatase (TNSALP).
