# Supplementary material for: Clinical Characteristics of Persistent Hypophosphatasemia Uncovered in Adult Patients: A Retrospective Study at a Japanese Tertiary Hospital
Source: J Clin Med. 2024 Nov 23;13(23):7078. doi: 10.3390/jcm13237078 (PMC11642156; doi:10.3390/jcm13237078)
Supplement: Supplementary file 1 [file jcm-13-07078-s001.zip › jcm-3333505-supplementary.pdf]

Table S1. Possible causes of known hypophosphatasemia.

Treatment-related causes	Disease-related causes	Other cause
Corticosteroids<br>Clofibrate<br>Antiresorptive drugs<br>Hormone therapy (selective estrogen receptor modulators)<br>Immunosuppressants<br>Chemotherapy<br>Surgery<br>Vitamin D intoxication<br>Massive infusion	Hypophosphatasia<br>Severe disorders including sepsis, renal failure, hepatic failure, multi-organ failure, and major trauma<br>Cancers<br>Hypoparathyroidism<br>Hypothyroidism<br>Hypercortisolism<br>Severe Malnutrition<br>Multiple Myeloma<br>Celiac disease<br>Deficiency of magnesium, vitamin C, vitamin B12<br>Severe Anemia<br>Wilson disease<br>Cleidocranial dysplasia<br>Osteogenesis imperfecta<br>Mseleni joint disease	Improperly collected specimen

Table S2. Diseases and number of cases that required corticosteroid use.

Diagnosis for corticosteroid use	Number
Systemic lupus erythematosus	9
ANCA-associated vasculitis	5
Sarcoidosis	5
Pemphigus vulgaris	5
Rheumatoid arthritis	4
Myasthenia Gravis	3
Autoimmune hemolytic anemia	2
Sjögren syndrome	2
mixed connective tissue disease	2
Systemic scleroderma	2
Dermatomyositis	1
Castleman's disease	1
Psoriasis	1

Cancer	5
Hormone Replacement Therapy	3
Eosinophilic gastroenteritis	1
Interstitial pneumonia	1
Facial nerve palsy	1
Adult-onset Still's disease	1
Immunosuppressive therapy in renal transplantation	1
Allergic bronchopulmonary mycosis	1

Table S3. Diseases and number of cases that required immunosuppressant use.

Diagnosis for immunosuppressant use	Number
ANCA-associated vasculitis	5
Rheumatoid arthritis	6
Sjögren syndrome	4
Systemic lupus erythematosus	9
mixed connective tissue disease	2
Dermatomyositis	1
Castleman's disease	1
Sarcoidosis	5
Pemphigus vulgaris	4
Systemic scleroderma	2
Psoriasis	1
Interstitial pneumonia	1
Adult-onset Still's disease	1
Immunosuppressive therapy in renal transplantation	1
